# More about the World's Babies

**Published:** 1889-01-12

**Authors:** 


### Babies.

*(Continued from p. 217.)*

### II.—MORE ABOUT THE WORLD'S BABIES.

What queer little mortals many of the children of savages are, with their dusky hides, or their brilliant paint, their little pot-bellies and hollow backs, their frizzled heads, and their bead-like glittering eyes. There are few, if any, of them quite black at first, only a sort of whitey-brown, their swarthiness deepening with their years. This is the case even with the blackest races of Africa. Some of the children of the uncivilized races represented in the great assembly we brought together in the last chapter are really very pretty. The Malay infants yonder, for instance, are some of them charming little creatures, and even the aboriginal Australians would many of them be winsome enough, in spite of their obtrusive stomachs and shock-heads, if only they were washed occasionally. They are washed sometimes, but it is only when they happen to tumble into the water or meet with some other accident which renders washing a matter of extreme necessity. Ordinarily they know as little about the process as do the Esquimaux, and it will easily be understood that the winsome ways of an Australian baby are best appreciated from a distance; closely observed, however, some cf their features are not bad, and they have a soft, half-wild, half-wistful expression of eye which is very attractive while they are quite young, but which soon gives place to the fierce, restless, suspicious gleam of the savage within. Australian mothers are remarkable for their affection for their children—so, at least, some travellers tell us. Their parental fondness is, indeed, carried to such excess of weakness as one may sometimes see in the drawing-rooms of civilized mamas, especially with the boys. They rarely or never correct them, and as a natural consequence the young rogues soon become utterly regardless of mother, whom they begin to illuse or tyrannize over before they are much higher than "six-pen'orth of ha'pence." An American writer, who ought to know something upon the subject, says that he likes to touch the babies, for their flesh is cool and tender; but that the most delicate texture is the skin of a black or mulatto baby. It is satiny, and fine. These children are always great pets, as everyone knows who has lived in the South. They awaken, he adds, with grim humour, a great deal of love in the whites till they become old enough to sell, and in their mothers somewhat longer than that. This, of course, was written before the American war put an end to bargains in babies.

Few, if any, of the infants in this vast gathering are black; but not a few of them are of a more or less fiery red. Yonder are the Kaffirs, for instance—daubed all over with brilliant red paint, which for many months is renewed as often as proper smartness may require. These coats of paint are the swaddling-clothes of the baby Kaffir. The baby Kaffir is never "short-coated."

Talking of children who never wear clothes reminds one that it is not only in hot climates that they are to be found. The Fuegians—inhabitants of a country in which even in the summer season people have been known to die of cold if they have ventured up a very little above the level of the sea—never give their infants any clothing. A naked Fuegian mother has been seen to stand quite unconcernedly nursing her baby while a driving storm has piled up the snow between her breast and her infant. We have, you will observe, a few of these little Fuegians in our great assembly—all of them, you see, as naked as they were born. Near them you may see a larger contingent of young Patagonians tucked up snugly in the cradles in which they are accustomed to be carried about by their mothers, or slung at the saddle-bows of the horses. They have to rough it, these young Patagonians, but it is not from any want of love in the mothers. The death of a child among them is marked by a display of sincere grief on the part of the parents. The horse it has been accustomed to travel on during their migrations from place to place is brought up, the gear placed on it, even to the cradle, and the horse, thus fully caparisoned, is strangled by means of lassoes. The saddle, gear, cradle, and all belonging to the child are burned, the women crying and singing. The parents, moreover, throw their own valuables into the fire, to express their grief. It is the custom also for chiefs and relatives to send gifts to the bereaved parents, to divert their minds from dwelling on their loss.

Their neighbours, the Araucanians, roll up their infants in bundles, and bandage arms and legs so that they cannot move, very much as they do in Germany and Sweden and many other parts of Europe. The quietness of the little mummies being secured—so far as legs and arms are concerned—they may be safely disposed of, pretty much as any other bundle may be. In Sweden, by the way, the mothers thus pack up their infants, and in winter, if they have nobody to leave them at home with when they want to go to Sunday service, they will carry them off to the door of the church, where they will leave them safely stowed away beneath a covering of snow, with just a little air-hole for breathing purposes, where they will lie perfectly safe and warm till service is over. The Araucanians do not put their babies in the snow, but they lay them in bamboo cradles, and hang them up on a nail in the wall of the house. Or they may be laid in baskets suspended from the ceiling, so as to permit of their being rocked to sleep by means of a cord. "These infants," says one writer, describing the Araucanians, "are perfect models of behaviour, allowing themselves to be hung on pegs without betraying any signs of life except the movement of the eyes. As soon as they can walk they run about without any clothes till they are seven or eight years old."

These are South Americans. The Indians of North America dispose of their babies after very much the same fashion, only their cradles are different. The North American cradle is a sort of chrysalis case, into which the child is put feet downwards, and in which it may be slung on the back or stood against the wall, or hung up on a branch of a tree. The infant's limbs are confined pretty much as though they were bandaged, and only the head is visible. These cradles are often very elaborately fashioned and richly coloured, the parents evidently taking as great a pleasure and pride in them as some other folks take in their children's cribs and bassinettes. Most people, probably, have read of the strong and pathetic show of affection for their babies common among the Red Indians of America. Should the baby die during its cradle days it is tenderly buried, and the disconsolate mother fills the cradle with a bundle of black quills and feathers, and carries it about with her wherever she goes, however long her journeys may be, or however hard or fatiguing the way. For a year and sometimes longer she never parts with it, and, says Catlin in his well-known account of his travels among these people, "she often lays or stands it leaning against the side of the wigwam when she is engaged in needlework, and chats and talks to it as familiarly and affectionately as though it were the loved infant." So tenderly attached to their infants are these mothers, and so vivid appears to be their imagination that they will be even more strict in their attention to the mourning-cradle than they are to their living infants.

"Whilst I remained among them, a couple, whose tent was adjacent to mine, lost their infant son. The parents were so much affected as to occasion the death of the father. The woman, who had hitherto been inconsolable, no sooner saw her husband expire than she dried up her tears, and appeared cheerful and resigned. As I knew not how to account for so extraordinary a transition, I took an opportunity to ask her the reason of it, telling her at the same time that I should have imagined the loss of her husband would have occasioned an increase of grief rather than such a sudden diminution of it.

"She informed me that as the child was so young when it died, and unable to support itself in the land of spirits, both she and her husband had been apprehensive that its situation would be far from being happy; but no sooner did she behold its father depart for the same place, who not only loved the child with the tenderest affection, but was a good hunter, and would be able to provide plentifully for its support, than she ceased to mourn. She now saw no reason to continue her tears, as the child, on whom she doted, was under the care and protection of a fond father, and she had only one wish that remained ungratified, which was that of being herself with them."

*(To be continued.)*

